# Efficacy and Safety of Ojeok-San Plus Saengmaek-San for Gastroesophageal Reflux-Induced Chronic Cough: A Pilot, Randomized, Double-Blind, Placebo-Controlled Trial

**DOI:** 10.3389/fphar.2022.787860

**Published:** 2022-03-01

**Authors:** Yee Ran Lyu, Kwan-Il Kim, Changsop Yang, So-Young Jung, O Jin Kwon, Hee-Jae Jung, Jun-Hwan Lee, Beom-Joon Lee

**Affiliations:** ^1^ Korean Medicine Science Research Division, Korea Institute of Oriental Medicine, Daejeon, South Korea; ^2^ Division of Allergy, Immune and Respiratory System, Department of Internal Medicine, College of Korean Medicine, Kyung Hee University, Seoul, South Korea; ^3^ Clinical Medicine Division, R&D Strategy Division, Korea Institute of Oriental Medicine, Daejeon, South Korea; ^4^ Korean Medicine Life Science, Campus of Korean Institute of Oriental Medicine, University of Science and Technology (UST), Daejeon, South Korea

**Keywords:** chronic cough, gastroesophageal reflux disease, herbal medicine, Ojeok-san, Saengmaek-san

## Abstract

**Introduction:** Gastroesophageal reflux-induced chronic cough (GERC) is one of the most common etiologies of chronic cough. Despite the growing prevalence and interest in GERC, no effective treatment is currently available. In our study, we used a combination of herbal medicines, *Ojeok-san* (OJS) plus *Saengmaek-san* (SMS), for the treatment of GERC.

**Methods:** We conducted a pilot, randomized, placebo-controlled, parallel-arm, single-center clinical trial to assess the feasibility of our study protocol, as our study is the first herbal medicine trial for GERC. All enrolled participants were randomly assigned to either the intervention or placebo group in a 1:1 ratio and were administered trial drugs three times a day for 6 weeks, with an evaluation visit performed every 2 weeks for their efficacy and safety assessment until the follow-up visit (week 8). We evaluated the severity and frequency of cough, cough-specific quality of life, airway hypersensitivity, and reflux-related gastrointestinal symptoms, as well as pattern identification, to investigate the complex mechanisms of reflux cough syndrome.

**Results:** A total of 30 participants were enrolled, and 25 completed the study at Kyung Hee University Korean Medicine Hospital from 26 December 2018 to 31 May 2021. OJS plus SMS significantly improved the cough diary score (CDS), cough visual analog scale, Korean version of the Leicester Cough Questionnaire, Hull Airway Reflux Questionnaire, and Gastrointestinal Symptom Rating Scale after the treatment compared to the baseline. Notably, OJS plus SMS showed significant efficacy in the daytime and total CDS compared with the placebo. Only one adverse event was observed during the trial, and no serious adverse events occurred. Additionally, we achieved successful results in feasibility outcomes by exceeding the ratio of 80%.

**Conclusion:** We confirmed the feasibility of our trial design and demonstrated the potential of OJS plus SMS in relieving the severity of cough and GI symptoms in GERC patients with safe and successful feasibility results. We anticipate that our study results will be used as the basis for further large-scale, well-designed, confirmatory trials to evaluate the safety and efficacy of OJS plus SMS in GERC.

**Clinical Trial Registration**: [https://cris.nih.go.kr], identifier WHO International Clinical Trials Registry Platform, Clinical Research Information Service [KCT0003115].

## 1 Introduction

Cough is the most common respiratory complaint for which patients seek medical care for various reasons. Based on duration, cough can be classified into three categories by which diagnosis and treatment are determined: acute, lasting <3 weeks; subacute, lasting 3–8 weeks; and chronic, lasting >8 weeks (Irwin and Madison, 2000). Acute cough, which is often caused by upper respiratory tract infection, is generally self-limiting and benign, whereas chronic cough has more complex problems, mostly attributed to multiple causes, and requires careful evaluation (Birring et al., 2003). It is also associated with various adverse psychosocial or physical effects on the quality of life by causing anxiety, physical discomfort, social isolation, nausea, chest pain, and urinary incontinence (French et al., 1998).

The major causes of chronic cough include gastroesophageal reflux disease (GERD), upper airway cough syndrome, and cough variant asthma, in patients with a normal chest radiograph and not on angiotensin-converting enzyme inhibitors (Chung and Pavord, 2008). Among them, there has been growing interest in gastroesophageal reflux-induced chronic cough (GERC), as the prevalence of GERD is increasing over time, and GERD and chronic cough are linked in a cause-and-effect relationship (Jinnai et al., 2008). Previous studies have reported that GERD accounts for 10%–40% of the causes of chronic cough, and its incidence is anticipated to increase worldwide (Morice and Kastelik, 2003). The mechanisms by which GERD induces cough are known to be involved in the stimulation of the esophageal-bronchial cough reflex mediated by the afferent nerves in the distal esophagus (reflex hypothesis) and microaspiration of the gastric contents into the airways (reflux hypothesis) (Irwin, 2006). Moreover, some studies reported that GERD and cough may aggravate each other, thereby leading to treatment refractoriness (Ing, 2004).

When GERD causes cough, gastrointestinal (GI) symptoms are not generally found for up to 75% of the time, and cough is the sole manifestation that often occurs in the daytime (Irwin et al., 1993). Thus, it is challenging to diagnose chronic cough due to GERD, and even the most sensitive and specific test for GERD, 24-h esophageal pH monitoring, has limitations in the diagnosis of reflux cough syndrome (Irwin, 2006). Therefore, the American College of Chest Physicians guidelines suggested using a diagnostic algorithm by excluding other potential chronic cough causes to predict reflux cough syndrome and only to use 24-h esophageal pH monitoring for those who have failed antireflux therapy or those who have a strong clinical suspicion of gastroesophageal reflux (Kahrilas et al., 2016). When patients with chronic cough fit the clinical profile for GERD, they were empirically administered antireflux medicines, including proton pump inhibitors (PPIs), H2-receptor antagonists, alginate, or antacid therapy. However, these anti-reflux drugs were found to be ineffective for patients without GI symptoms and were only recommended for those with heartburn and regurgitation (Xu et al., 2016). For patients who have no GI symptoms such as heartburn, which accounts for the majority of patients with reflux-cough, no specific therapy has been recommended yet, and only some of the lifestyle changes, such as diet modification or elevation of the head of their beds, have been suggested (Kahrilas et al., 2016). Therefore, an effective therapeutic agent for GERD-induced chronic cough, both in patients with and without GI syndromes, is required.

In this study, we focused on herbal medicines that have been used for centuries in East Asian countries to treat diverse diseases, including GERD and cough. These herbal medicines have often been used as a combination to manage comorbidities, such as GERD-induced cough. Moreover, there are 56 types of insurance-covered Korean medicine (KM) granules that are frequently prescribed in clinics in Korea. Thus, we chose *Ojeok-san* (OJS) and *Saengmaek-san* (SMS) among them to make a combination of herbal medicines for our study, as OJS is one of the most frequently prescribed insurance-covered KM granules for digestive disorders, and SMS is also widely used to relieve cough.

OJS, comprising 17 herbs, was originally recorded in an ancient Korean medicinal book named “Donguibogam.” OJS has been widely used to treat digestive disorders, including GERD, chronic gastroenteritis, stomach cramps, low back pain, neuralgia, and common cold (Lee et al., 2008). A recent study also reported the effects of OJS on airway inflammation and pulmonary fibrosis (Shin et al., 2013). SMS, a prescription consisting of three herbs, Liriopis Tuber, Ginseng Radix, and Schisandrae Fructus, has been primarily used for dry cough by moisturizing the respiratory mucosa. It has been previously reported to treat cardiovascular and neurological disorders (Lai-Hong et al., 2013; Chiang et al., 2021), and recently, SMS was shown to regulate GI motility by increasing the activity of Cajal cells in the GI tract (Kim, 2013). The combination of these two herbal medicines, OJS, and SMS, has been used in clinics to treat GERC, as an indication for digestive and respiratory diseases. In a previous study, we also reported cases of GERD-induced chronic cough treated with OJS plus SMS. We anticipate that this combination of OJS and SMS will be effective in GERC and be economical for patients with chronic cough, as both of these drugs are insurance-covered granules.

In our study, we aimed to explore the efficacy and safety of OJS plus SMS for patients with chronic cough due to GERD. As our study is the first herbal medicine trial for GERD-induced cough, we designed it as a pilot, randomized, placebo-controlled, parallel-arm, single-center clinical trial. We evaluated the severity and frequency of cough, cough-specific quality of life, airway hypersensitivity, and reflux-related GI symptoms to investigate the complex mechanisms of reflux cough syndrome. Our study provides preliminary information about the feasibility of the planned design, the number of eligible patients, the duration required for patient recruitment, and appropriate outcome measures for our next large-scale confirmatory trial.

## 2 Materials and Methods

### 2.1 Study Design

This study is a pilot, randomized, placebo-controlled, parallel-arm, single-center clinical trial to investigate the feasibility of the study protocol, interventions, and outcome measures for further large-scale clinical trials of OJS plus SMS in patients with GERD-induced chronic cough. The trial was conducted at Kyung Hee University Korean Medicine Hospital from 26 December 2018 to 31 May 2021. A total of 30 participants, originally planned, were enrolled, and 25 completed the trial without violating the protocol. All participants who voluntarily signed a written consent form were evaluated for their eligibility based on inclusion and exclusion criteria at screening, and they were asked to record their daily cough symptoms during the run-in period of 7 days. Those who recorded cough diary more than 10 times and scored over an average of two in cough diary score (CDS) were finally enrolled in this trial. All of the enrolled participants were randomly assigned to either the intervention (OJS plus SMS 5.76 g) or placebo group in a 1:1 ratio, and they were administered trial drugs three times a day for 6 weeks. All participants visited the hospital every 2 weeks for their efficacy and safety evaluation until the follow-up period as follows: week 0 (baseline), 2, 4, 6, and 8 (follow-up). More detailed information regarding the trial is described in a previously published protocol (Bhang et al., 2020).

This study was conducted in accordance with the Declaration of Helsinki and Good Clinical Practice guidelines and followed the guidelines of Consolidated Standards of Reporting Trials guidelines and Reporting Randomized Controlled Trials of Herbal Interventions (Cheng et al., 2017). The study protocol was authorized by the Ministry of Food and Drug Safety of Korea (MFDS) (approval number 31617) and approved by the Institutional Review Board of the Kyung Hee University Korean Medicine Hospital (KOMCIRB 2018-05-017-001), which was registered at the Clinical Research Information Service (KCT0003115). All participants provided informed consent before enrollment, and all data were coded and kept confidential.

### 2.2 Participants

#### 2.2.1 Inclusion Criteria

Participants who met the following criteria were included in this trial: (1) aged between 19 and 70 years, (2) a history of cough continuously for >8 weeks, (3) diagnosed with GERD within the past year, and (4) patients who consent to participate.

#### 2.2.2 Exclusion Criteria

Participants were excluded for any of the following reasons: (1) Present with abnormal findings, as established by chest radiograph, pulmonary function test (PFT) with bronchodilator test, fractional exhaled nitric oxide (FeNO), and nasal endoscopy, that might lead to cough; (2) Diagnosed with acute respiratory diseases (including upper respiratory tract infection) within the past month; (3) Diagnosed with chronic respiratory diseases (including bronchiectasis, bronchial asthma, interstitial lung disease, chronic obstructive pulmonary disease, and other chronic respiratory diseases) within the last 2 years; (4) Diagnosed with Los Angeles classification system grade C or higher GERD within the past year; (5) Exhibit symptoms indicative of malignant disease within the GI tract (severe dysphagia, bleeding, weight loss, anemia, bloody stools); (6) History of surgical or endoscopic antireflux treatment; (7) Currently have a disorder such as blood-clotting disorder, ostnasal drip syndrome, or an active infection requiring systemic antibiotic therapy; (8) Have a lifetime smoking history of ≥20 packs (400 cigarettes); (9) Have used an angiotensin-converting enzyme inhibitor during the previous 4 months; (10) Have used cough medicines, glucocorticoids, leukotriene receptor antagonists, anticholinergic drugs, long-acting β2-agonists, antihistamines, PPIs, histamine receptor antagonists, mucosa-protective agents, GI motility promoters, antacids, antidepressants, anxiolytics, lower esophageal sphincter agonists, or any herbal medication within the previous 2 weeks; (11) Have allergies or sensitivities to the experimental medicine/placebo; (12) Have a body mass index <18.5 kg/m2; (13) Have an aspartate aminotransferase (AST) or alanine aminotransferase (ALT) level at least, two fold higher than the upper limit of normal or a serum creatinine level at least 1.2-fold the upper limit of normal; (14) Have a mean CDS <2 during the 1-week run-in period; (15) Record <10 entries in the cough diary during the 1-week run-in period; (16) Have a history of malignant tumors (lung or esophageal cancer) within the last 5 years; (17) Are excessive drinkers; (18) Are pregnant or breastfeeding; (19) Do not consent to use birth control during the trial; (20) Have participated in clinical trials for the same disease within the past 3 months; and (21) Are deemed unsuitable by the investigators.

### 2.3 Randomization and Blinding

Randomization (1:1 allocation) was conducted by an independent statistician using a randomization table created by SAS version 9.1.3 software (SAS Institute Inc., Cary, NC, United States). The manufacturer provided the clinical trial drugs labeled with the participant’s identification code on the packages based on the randomization list, and the management pharmacist supplied the drug to each participant according to the labeling. All participants and investigators were blinded throughout the trial using this strategy.

### 2.4 Intervention

The intervention drug, OJS plus SMS, is composed of 4.35 g of OJS and 1.41 g of SMS granules making it a total dose of 5.76 g, and each ingredient is described in Table 1 (Bhang et al., 2020). Each granule was approved by the MFDS, and the dosage was determined according to the approved dosage of the MFDS. The placebo drug, comprising 4.35 g of OJS placebo and 1.41 g of SMS placebo, does not contain any of the active ingredients in OJS and SMS and is composed of ingredients including starch, lactose, citric acid, caramel color, and ginseng flavor powder. Both intervention and placebo drugs were matched in terms of appearance, taste, smell, and package and were provided by Han Kook Shin Yak Pharm Co. Ltd. (Nonsan, Chungnam, South Korea), a company that has obtained authorization from the Korea Good Manufacturing Practice. The clinical trial drugs were stored at the clinical research pharmacy in the Korean Medical Clinical Trial Center of Kyung Hee University Korean Medicine Hospital, and an independent well-trained pharmacist was responsible for all the procedures related to the drugs.

**TABLE 1:** Composition of Ojeok-san and Saengmaek-san.

Latin name	Amount (g)
Ojeok-san	
Atractylodis Rhizoma	0.95
Ephedrae Herba	0.2
Citri Unshius Pericarpium	0.4
Magnoliae Cortex	0.08
Platycodonis Radix	0.43
Aurantii Immaturus Fructus	0.31
Angelicae Gigantis Radix	0.37
Zingiberis Rhizoma	0.22
Paeoniae Radix	0.27
Poria Sclerotium	0.02
Cnidii Rhizoma	0.3
Angelicae Dahuricae Radix	0.31
Pinelliae Tuber	0.22
Cinnamomi Cortex	0.04
Glycyrrhizae Radix et Rhizoma	0.2
Zingiberis Rhizoma Recens	0.03
Total	4.35
Saengmaek-san	
Liriopis Tuber	0.75
Ginseng Radix	0.30
Schisandrae Fructus	0.36
Total	1.41

### 2.5 Efficacy Outcome Measures

The primary outcome was the change in CDS after 6 weeks of treatment compared to the baseline between the intervention and placebo groups. CDS is a patient-report outcome measure, including the severity and frequency of cough, each of which ranges from 0 to 4, with a maximum total score of eight points (Ours et al., 1999). A higher score indicated more severe and more frequent cough symptoms. Participants were required to record their CDS twice per day during the daytime and nighttime in the given cough diary, and the total CDS was calculated by averaging the daytime and nighttime CDS. As severity and frequency are the most widely used components for assessing cough (Birring and Spinou, 2015), we selected the CDS for our primary measures in chronic cough patients.

For secondary outcome measures, the severity of cough, cough-specific health-related quality of life (HRQL), airway reflux symptoms, and GI symptoms were assessed in all participants during the trial period.

As an additional cough severity scale, the cough visual analog scale (VAS) was evaluated, which can be scored from 0 to 100 points, with indicating 0 as “no cough” and 100 indicating “unbearable cough.” It is simple but one of the most widely used instruments for assessing cough, which has been reported to be highly responsive when used as an outcome measure in clinical studies of patients with chronic cough (Raj and Birring, 2007). Participants recorded the cough VAS daily in their cough diary, and we calculated the average of the previous 2-weeks score to estimate the effects of intervention between groups.

The cough-specific HRQL was assessed using the Korean version of the Leicester Cough Questionnaire (LCQ-K) to determine the differences in chronic cough patients. The LCQ-K is composed of 19 items, which are divided into three parts: physical, mental, and social. Each item score ranges from one to seven points, with higher scores indicating better quality of life-related to chronic cough (Birring et al., 2003). It is the most frequently used measure of HRQL in patients with chronic cough and has been validated in the Korean version (Han et al., 2014).

Participants’ airway reflux symptoms were also measured using the Hull Airway Reflux Questionnaire (HARQ), which is a self-assessment tool developed to diagnose and evaluate airway hypersensitivity due to laryngopharyngeal reflux. It consists of 14 questions, asking about the severity of concomitant symptoms, cough triggers, and exacerbation factors; each item ranges from 0 to 5, with a total score of 70 points. The higher the score, the more severe the airway reflux symptoms. As patients with chronic cough are often reported to have hypersensitivity of their cough reflex, particularly when caused by GERD, it is important to observe symptoms related to airway reflux in GERD induced cough patients (Morice et al., 2011).

GI symptoms were assessed using the Korean Gastrointestinal Symptom Rating Scale (K-GSRS) every 2 weeks. GSRS is a validated self-report GI symptom scale that is widely used worldwide for patients with both upper and lower GI symptoms. It has been validated in GERD, irritable bowel syndrome, and peptic ulcer disease (Revicki et al., 1997). The K-GSRS was developed by adding one symptom item to the originally validated GSRS, containing a total of 16 symptom items, which are divided into five symptom scales of reflux, indigestion, abdominal pain, constipation, and diarrhea. Each item is scored by a 5-point Likert scale, in which “1” indicates absence and “5” the higher frequency or intensity of the symptoms (Kwon et al., 2008).

Additionally, we evaluated Pattern Identification for both symptoms of chronic cough (Kim et al., 2015) and GERD (Han et al., 2015) using each developed questionnaire. As pattern identification is an important diagnostic and treatment tool in traditional KM, we used these two pattern identification questionnaires to observe the distribution of patterns in patients with GERC and to distinguish which patterns of participants respond best to our intervention and whether the pattern changes after the treatment.

### 2.6 Safety Outcomes

Safety was assessed at every trial visit by recording adverse events (AEs) and vital signs (blood pressure, pulse, and body temperature). Additionally, laboratory examinations of liver function tests, including AST, ALT, alkaline phosphatase, gamma-glutamyl transpeptidase (γ-GTP), total bilirubin, blood urea nitrogen (BUN), and creatinine, were performed at baseline and after 6 weeks to evaluate whether traditional herbal medicines affect the liver and renal functions.

### 2.7 Sample Size and Statistical Analysis

The sample size in our study was determined based on a previous study that recommended sample size of 12 per group for pilot studies when there was no prior evidence to calculate (Julious, 2005). Considering a dropout rate of 20%, we decided to enroll 30 participants for our pilot study. Based on this pilot study, the sample size needed for a large-scale confirmatory trial will be estimated.

Statistical analysis was performed by an independent professional statistician using SAS® (version 9.4, SAS Institute, Cary, NC, United States). All data analyses were primarily based on intention-to-treat (ITT) analysis. When a missing value was observed, a multiple imputation method was used in efficacy outcome measures. Continuous variables are presented as mean (95% confidence interval), and categorical variables are reported as frequencies (percentages). Significance was accepted at a two-sided test with an α-level of 0.05. For efficacy outcome measures, analysis of covariance (ANCOVA) was performed.

## 3 Results

### 3.1 Study Participants and Baseline Characteristics

**FIGURE 1:**
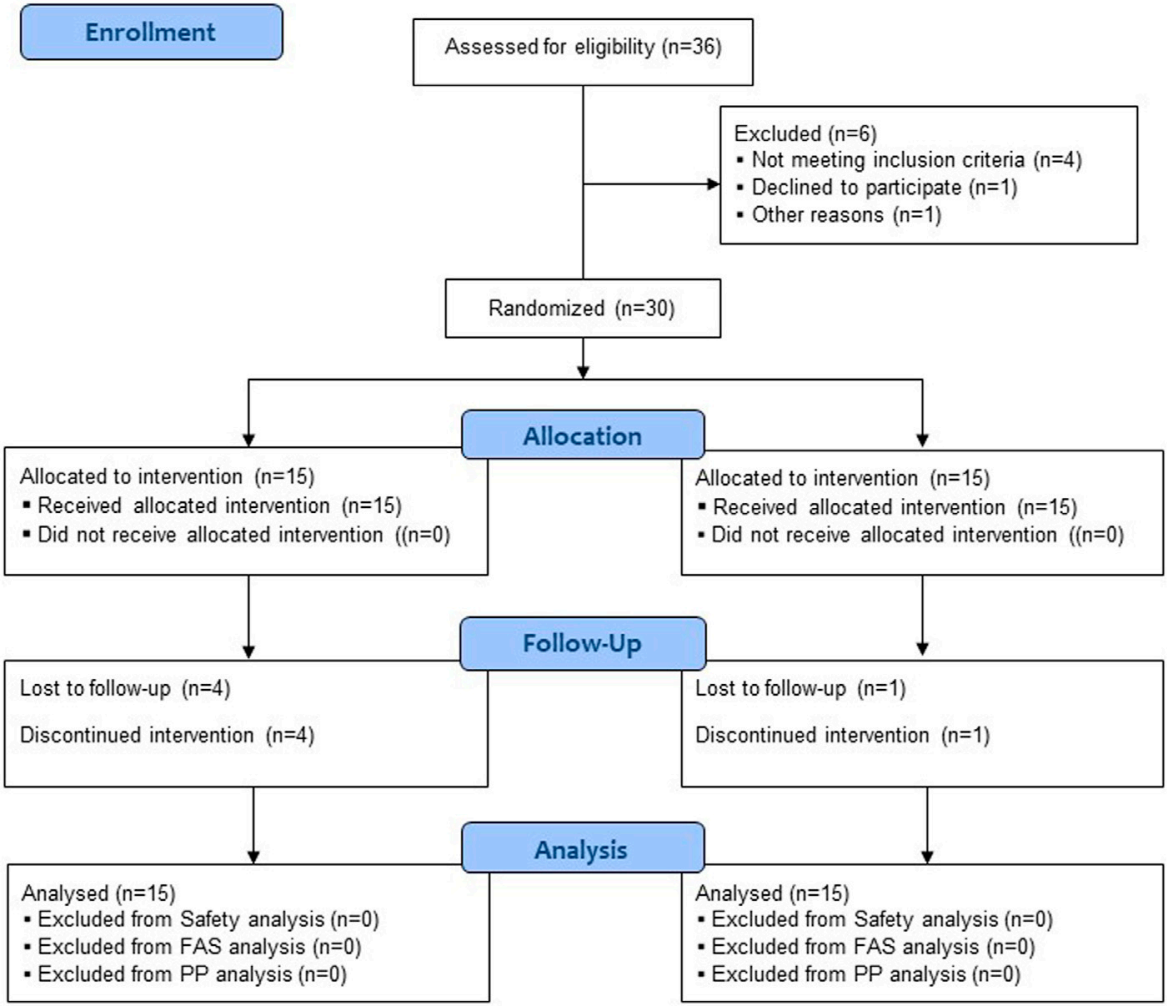
Flow chart for the study subjects.

A total of 36 participants were screened, and six were excluded as they did not meet the inclusion/exclusion criteria or declined to participate. Among the 30 enrolled participants, 25 completed the trial, and five dropped out due to the withdrawal of informed consent and the occurrence of AE (Figure 1). All 30 participants were included in the safety and full analysis set analysis.

The baseline characteristics of the study participants are shown in Table 2. There were no significant differences in age, sex, height, weight, body mass index, drinking, duration of symptoms, vital signs, pattern identification, and outcome measures of CDS and VAS between the intervention and control groups, except for the diastolic blood pressure (DBP) and pulse. The results of DBP and pulse in the placebo group were lower than those in the intervention group; however, both were determined as not clinically significant by investigators.

**TABLE 2:** Baseline characteristics.

Characteristic	OJS plus SMS (*n* = 15)	Placebo (*n* = 15)	p-value
Age (year)^a^	40.47 (31.81, 49.12)	39.13 (31.14, 47.13)	0.8101
Gender (M/F)^b^	8 (53.33%)/7 (46.67%)	7 (46.67%)/8 (53.33%)	0.9999
Height (cm)^a^	169.5 (164.3, 174.8)	169.3 (165.4, 173.2)	0.9341
Weight (kg)^a^	74.55 (64.56, 84.54)	68.38 (60.36, 76.40)	0.3106
BMI (kg/m^2^)^a^	25.73 (23.05, 28.41)	23.70 (21.57, 25.83)	0.2148
Drinking (Yes/No)^b^	4 (26.67%)/11 (73.33%)	3 (20.00%)/12 (80.00%)	0.9999
Duration of symptom (month)^a^	40.93 (25.93, 55.94)	36.00 (21.69, 50.31)	0.6138
Vital sign^a^			
SBP	123.6 (117.6, 129.6)	118.3 (112.8, 123.8)	0.1747
DBP	80.60 (74.18, 87.02)	72.13 (67.49, 76.78)	0.0296*
PULSE	85.67 (79.13, 92.20)	74.67 (69.75, 79.59)	0.0075**
Temp	36.54 (36.49, 36.59)	36.56 (36.51, 36.61)	0.5341
CDS^a^	3.91 (3.44, 4.37)	4.18 (3.46, 4.90)	0.4996
VAS^†^	40.29 (32.35, 48.22)	42.58 (29.04, 56.12)	0.7563
Pattern Identification for GERD^b^			
Stagnation of the Liver Qi	10 (66.67%)	9 (60.00%)	0.6838
Stomach yin deficiency	2 (13.33%)	2 (13.33%)	
Spleen-stomach weakness	3 (20.00%)	2 (13.33%)	
Spleen-stomach dampness-heat	0 (0.00%)	2 (13.33%)	
Pattern Identification for Chronic Cough^b^			
Wind-cold	5 (33.3%)	6 (40.0%)	0.3018
Phlegm turbidity	3 (20.0%)	1 (6.7%)	
Fire-heat	6 (40.0%)	3 (20.0%)	
Lung deficiency	1 (6.7%)	2 (13.3%)	
Kidney yang deficiency	0 (0.0%)	3 (20.0%)	

a Student`s independent *t*-test.

b Fisher`s exact test (*p < 0.05, **p < 0.01).

### 3.2 Primary Outcome Measurement

**FIGURE 2:**
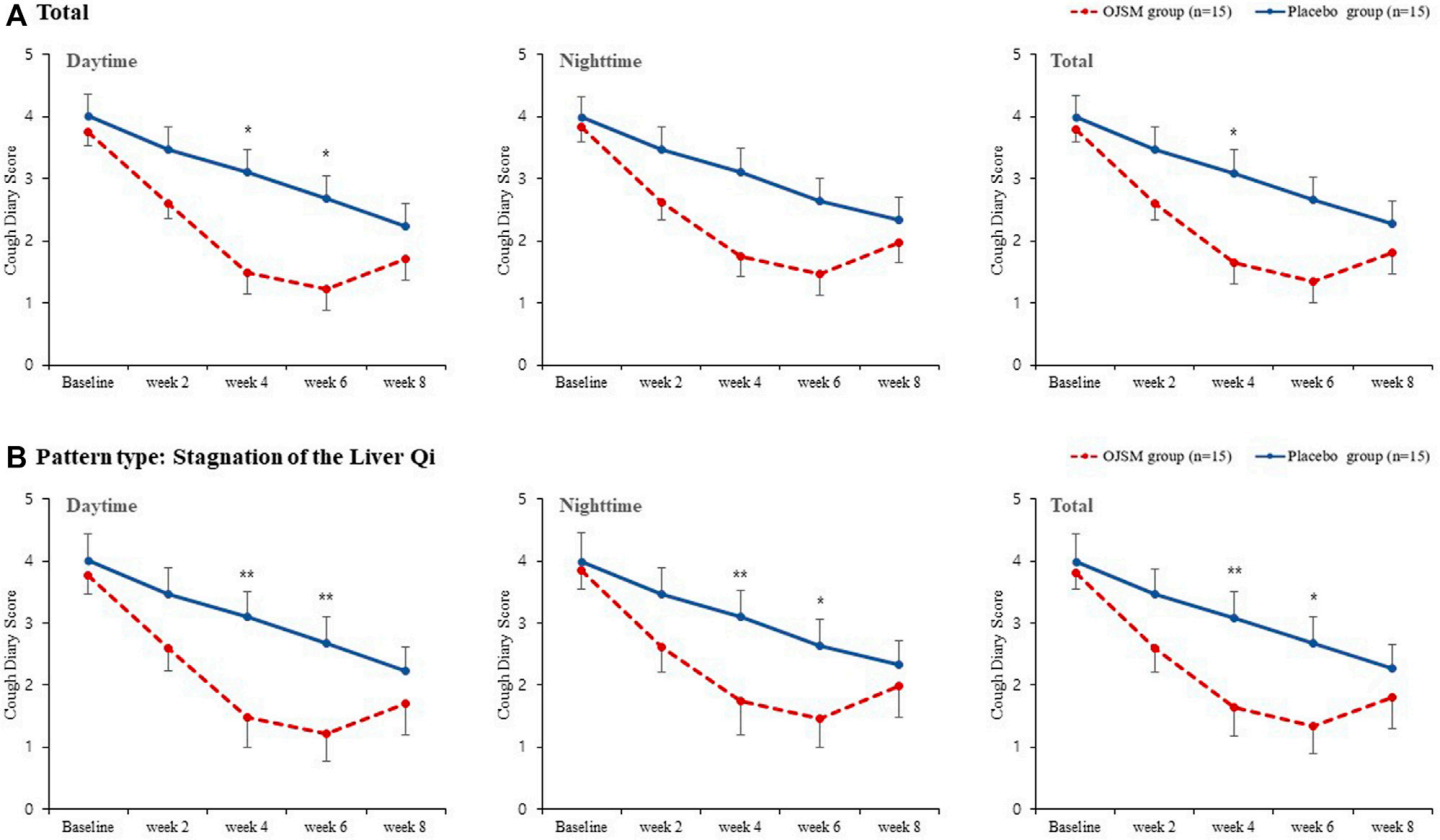
Cough diary score in OJS plus SMS group and placebo group. Data were presented as mean ± SE (*p < 0.05, **p < 0.01, and ***p < 0.001). **A** Total; **B** Stagnation of Liver Qi.

The primary outcome measure, CDS, gradually decreased from baseline to the end of treatment (week 6) in both the intervention and placebo groups. In OJS plus SMS group, the total CDS score was 3.91 in baseline and decreased to 2.75 in week 2, 1.80 in week 4, and 1.57 in week 6 with significant changes. However, it slightly increased to 1.90 in week 8 compared to week 6, although all showed statistical improvement compared to the baseline. In the placebo group, the total CDS score also significantly decreased from 4.18 at baseline, to 3.22, 2.83, 2.51, and 2.07 at week 2, 4, 6, and 8, respectively within the group. When comparing differences between the OJS plus SMS and placebo groups, the total CDS scores were shown to be improved more in the OJS plus SMS group than in the placebo group, with the statistical differences shown in week four between groups (*p* = 0.0427) (Figure 2, Supplementary Table S1).

In the subscale analysis of daytime and nighttime CDS scores, the intervention and control groups showed significant improvements within groups in both daytime and nighttime. In particular, the OJS plus SMS group showed a significant decrease in daytime cough symptoms compared with the placebo group at weeks four and 6 in the daytime (*p* = 0.0191, 0.0267, respectively). In the nighttime CDS score, the participants administered OJS plus SMS showed better improvements than the placebo group, although the difference was not statistically significant.

Additionally, CDS was analyzed for participants who belonged to the pattern type of stagnation of the Liver Qi, comprising 10 participants in the OJS plus SMS group and nine in the placebo group. We found that OJS plus SMS had significant efficacy in the daytime, night time, and total CDS compared to the placebo in weeks 4 and 6, and showed greater improvements than the results from all participants.

### 3.3 Secondary Outcome Measurement

#### 3.3.1 Cough Visual Analog Scale

OJS plus SMS showed a significant decrease in cough VAS after 2, 4, 6, and 8 weeks compared to the baseline. The statistical improvements in cough severity were shown immediately after week 2, which had decreased by 12.86 points compared to the baseline. After treatment for 4 weeks, the cough VAS decreased by 24.89 points, which was the lowest among the whole trial period. The score slightly increased in weeks 6 and 8, as shown in Table 3.

**TABLE 3:** Cough VAS in OJS plus SMS group and placebo group.

Cough VAS	OJS plus SMS (*n* = 15)		Placebo (*n* = 15)		*P* value^a^ (OJS + SMS *versus* placebo)
	Mean (95% CI)	*P* value^b^ (within group)	Mean (95% CI)	*P* value^b^ (within group)	
Baseline	40.29 (32.35, 48.22)		42.58 (29.04, 56.12)		
Week 2	27.43 (19.08, 35.78)	0.0011**	32.52 (19.49, 46.55)	0.0106*	0.4654
Week 4	15.39 (7.32, 23.46)	<0.0001***	27.01 (14.53, 39.48)	0.0029**	0.0752
Week 6	16.62 (6.72, 26.52)	<0.0001***	25.52 (14.57, 36.47)	<0.0001***	0.1937
Week 8	18.53 (8.32, 28.74)	<0.0001****	18.84 (8.74, 28.94)	<0.0001***	0.8749

a p-value by Analysis of covariance (ANCOVA); OJS, Ojeok-san; SMS, Saengmaek-san; VAS, visual analogue scale; Data are presented as mean (95% CI) (*p < 0.05, **p < 0.01, and ***p < 0.001.).

b p-value by paired *t*-test.

The cough VAS score also showed significant improvements when treated with a placebo at weeks 2, 4, 6, and 8. When compared with the results from the OJS plus SMS group, the cough VAS score showed a steeper decline in the OJS plus SMS group than in the placebo group until week 6, since it decreased by 23.67 in the OJS plus SMS group, while by 17.06 in the placebo group at week 6. However, the score did not differ between groups after 8 weeks, and there were no statistical differences between the groups at every visit.

#### 3.3.2 Leicester Cough Questionnaire–Korean Version

The total LCQ-K score improved significantly from week two to week eight compared to the baseline in the OJS plus SMS group. It had gradually increased during the treatment period, as scored 16.95 at week 2, 17.45 at week 4, and 18.02 at week 6 compared to the baseline score of 14.75. The score was also increased in the placebo group, although the changes in each visit were not as high as those in the OJS plus SMS group until week 6. The differences between the groups were not statistically significant.

In the subscale analysis, the results of physical, psychological, and social scales also showed similar results. Both OJS plus SMS and placebo improved significantly throughout the trial period compared to the baseline, except for the social scale in week eight in the intervention group, and there were no significant differences between the two groups. On a physical and social scale, the OJS plus SMS showed more improvement than placebo until week 6 and then reversed at week 8. Only on the psychological scale, OJS plus SMS showed better quality of life than placebo throughout the whole trial period, including the follow-up visit (Table 4).

**TABLE 4:** Leicester cough questionnaire in OJS plus SMS group and placebo group.

LCQ	OJS plus SMS (*n* = 15)		Placebo (*n* = 15)		*P* value^a^ (OJS + SMS *versus* placebo)
	Mean (95% CI)	*P* value^b^ (within group)	Mean (95% CI)	*P* value^b^ (within group)	
Physical					
Baseline	4.72 (4.42, 5.01)		4.43 (3.98, 4.88)		
Week 2	5.43 (5.04, 5.83)	<0.0001***	5.12 (4.76, 5.48)	0.0034**	0.4779
Week 4	5.56 (5.19, 5.94)	<0.0001***	5.27 (4.92, 5.63)	<0.0001***	0.5605
Week 6	5.79 (5.34, 6.24)	<0.0001***	5.42 (4.99, 5.85)	<0.0001***	0.4478
Week 8	5.39 (4.93, 5.85)	0.0036**	5.41 (4.95, 5.87)	<0.0001***	0.4884
Psychological					
Baseline	4.84 (4.40, 5.27)		4.70 (4.06, 5.33)		
Week 2	5.43 (5.07, 5.80)	0.0012**	5.01 (4.48, 5.54)	0.0392*	0.1033
Week 4	5.62 (5.13, 6.11)	0.0087**	5.28 (4.72, 5.84)	0.0014**	0.4027
Week 6	5.90 (5.45, 6.36)	0.0004***	5.58 (5.07, 6.08)	<0.0001***	0.3725
Week 8	5.46 (4.90, 6.03)	0.0433*	5.23 (4.62, 5.84)	0.0029**	0.6977
Social					
Baseline	5.20 (4.69, 5.71)		4.70 (4.03, 5.37)		
Week 2	6.02 (5.61, 6.43)	<0.0001***	5.47 (4.88, 6.06)	0.0049**	0.3313
Week 4	6.13 (5.69, 6.57)	0.0002***	5.75 (5.17, 6.33)	<0.0001***	0.8112
Week 6	6.29 (5.82, 6.76)	<0.0001***	5.71 (5.12, 6.30)	<0.0001***	0.4127
Week 8	5.81 (5.19, 6.43)	0.0756	5.72 (5.04, 6.39)	<0.0001***	0.5187
Total					
Baseline	14.75 (13.61, 15.89)		13.83 (12.19, 15.47)		
Week 2	16.95 (15.85, 18.04)	<0.0001***	15.59 (14.27, 16.91)	0.0015**	0.2392
Week 4	17.45 (16.25, 18.64)	<0.0001***	16.29 (14.84, 17.75)	<0.0001***	0.4854
Week 6	18.02 (16.68, 19.36)	<0.0001***	16.76 (15.26, 18.26)	<0.0001***	0.4818
Week 8	16.76 (15.21, 18.32)	0.0106*	16.36 (14.61, 18.11)	<0.0001***	0.784

a p-value by Analysis of covariance (ANCOVA); OJS, Ojeok-san; SMS, Saengmaek-san; LCQ, leicester cough questionnaire; Data are presented as mean (95% CI) (*p < 0.05, **p < 0.01, and ***p < 0.001).

b p-value by paired *t*-test.

#### 3.3.3 Hull Airway Reflux Questionnaire3

The HARQ score in the OJS plus SMS group showed significant improvements at weeks 2, 4, and 8. The score decreased from 24.27 at baseline, to 17.19, 15.99 at week 2, and 4, respectively; however, increased to 20.22 at week 6, and decreased sharply again to 12.67 at week 8.

In the placebo group, the HARQ score decreased from 31.33 at baseline to 25.71 at week 2, which was not a significant change. It then significantly decreased to 17.13, 17.29, and 16.63 at week 2, 4, and 8, respectively, all found to be statistically significant compared to the baseline. No differences in HARQ scores were found between the groups during the trial period (Table 5).

**TABLE 5:** Hull Airway Reflux Questionnaire in OJS plus SMS group and Placebo group.

HARQ	OJS plus SMS (*n* = 15)		Placebo (*n* = 15)		*P* value^a^ (OJS + SMS *versus* placebo)
	Mean (95% CI)	*P* value^b^ (within group)	Mean (95% CI)	*P* value^b^ (within group)	
Baseline	24.27 (20.03, 28.50)		31.33 (23.82, 38.85)		
Week 2	17.19 (11.57, 22.81)	0.0012**	25.71 (18.13, 33.29)	0.1199	0.2832
Week 4	15.99 (9.49, 22.48)	0.0031**	17.13 (12.29, 21.97)	<0.0001***	0.6153
Week 6	20.22 (9.80, 30.63)	0.4201	17.29 (11.73, 22.85)	0.0001***	0.3398
Week 8	12.67 (7.28, 18.06)	<0.0001***	16.63 (9.03, 24.23)	<0.0001***	0.9367

a p-value by Analysis of covariance (ANCOVA); OJS, Ojeok-san; SMS, Saengmaek-san; HARQ, hull airway reflux questionnaire; Data are presented as mean (95% CI) (*p < 0.05, **p < 0.01, and ***p < 0.001).

b p-value by paired *t*-test.

#### 3.3.4 Gastrointestinal Symptom Rating Scale

For the assessment of GI symptoms in patients with GERC, the symptoms of reflux, abdominal pain, indigestion, constipation, and diarrhea were evaluated, although the direct association was assumed to be mostly based on symptoms of reflux. The OJS plus SMS gradually improved the symptoms of reflux compared to the baseline, which scored 55.00 at baseline, increased to 61.12, 69.46, 74.47, and 74.24 at week 2, 4, 6 and 8 respectively. Significant differences were found at weeks 6 and 8. In the placebo group, the reflux symptom score was 49.17 at baseline and increased to 60.00, 63.44, 68.69, and 72.15 at week 2, 4, 6, and 8, respectively, showing significant differences from week 4. The mean differences between the two groups were not detected, although we found that OJS plus SMS had improved reflux symptoms than placebo from week 4.

In the symptom score of abdominal pain, both the intervention and placebo groups demonstrated significant improvements after 2 weeks of treatment, although no differences were observed between the groups. When evaluated with the symptoms of indigestion, OJS plus SMS had significantly improved from week 6 and placebo from week 4, and also showed no statistical differences between groups. On the constipation scale, only the placebo group showed significant changes at weeks 6 and 8, and similar results were observed on the diarrhea scale; only the placebo group showed significant improvement at week 8 (Table 6).

**TABLE 6:** Gastrointestinal symptom rating scale in OJS plus SMS group and placebo group.

GSRS	OJS plus SMS (*n* = 15)		Placebo (*n* = 15)		*P* value^a^ (OJS + SMS *versus* placebo)
	Mean (95% CI)	*P* value^b^ (within group)	Mean (95% CI)	*P* value^b^ (within group)	
Reflux					
Baseline	55.00 (44.93, 65.07)		49.17 (38.55, 59.78)		
Week 2	61.12 (49.36, 72.89)	0.3483	60.00 (45.86, 74.14)	0.0723	0.7735
Week 4	69.46 (59.20, 79.72)	0.0514	63.44 (54.22, 72.67)	0.0172*	0.4323
Week 6	74.46 (61.19, 87.74)	0.0298*	68.69 (58.25, 79.14)	0.0051**	0.4736
Week 8	74.24 (65.06, 83.42)	0.0033**	72.15 (65.09, 79.22)	<0.0001***	0.8567
Abdominal pain					
Baseline	60.55 (52.10, 68.99)		53.89 (45.36, 62.41)		
Week 2	71.95 (62.81, 81.09)	0.0080**	60.55 (51.92, 69.18)	0.0409*	0.1598
Week 4	75.81 (66.49, 85.14)	0.0001***	70.07 (62.19, 77.95)	<0.0001***	0.8416
Week 6	75.74 (64.12, 87.36)	0.0115*	73.41 (63.58, 83.25)	0.0006***	0.9822
Week 8	82.31 (73.51, 91.11)	<0.0001***	76.03 (67.64, 84.42)	<0.0001***	0.6925
Indigestion					
Baseline	63.00 (52.18, 73.82)		52.67 (45.20, 60.13)		
Week 2	68.92 (59.66, 78.17)	0.095	58.67 (50.13, 67.20)	0.2066	0.3849
Week 4	70.90 (61.52, 80.28)	0.0513	59.29 (51.21, 67.37)	0.0366**	0.3569
Week 6	74.23 (63.90, 84.56)	0.0294*	67.99 (59.09, 76.90)	0.0032**	0.7875
Week 8	74.14 (63.81, 84.47)	0.0235*	70.11 (60.88, 79.35)	0.0007***	0.8749
Diarrhea					
Baseline	85.56 (77.29, 93.83)		78.33 (68.80, 87.85)		
Week 2	84.91 (77.83, 92.00)	0.8813	75.57 (67.87, 83.26)	0.5216	0.1465
Week 4	83.11 (74.96, 91.27)	0.6583	83.05 (76.40, 89.69)	0.1527	0.6712
Week 6	82.23 (73.14, 91.33)	0.5733	83.34 (74.62, 92.07)	0.2654	0.6029
Week 8	82.48 (75.19, 89.77)	0.4358	87.33 (79.88, 94.79)	0.0361*	0.1045
Constipation					
Baseline	81.68 (73.27, 90.09)		77.78 (69.47, 86.09)		
Week 2	81.79 (72.54, 91.04)	0.9632	76.11 (66.42, 85.80)	0.3828	0.6099
Week 4	83.98 (76.41, 91.54)	0.4028	82.85 (77.09, 88.62)	0.0923	0.7184
Week 6	85.61 (76.56, 94.66)	0.2215	85.26 (77.81, 92.72)	0.0282*	0.5842
Week 8	83.14 (75.21, 91.07)	0.6497	86.29 (78.92, 93.66)	0.0418*	0.2622

a p-value by Analysis of covariance (ANCOVA); OJS, Ojeok-san; SMS, Saengmaek-san; GSRS, gastrointestinal symptom rating scale; Data are presented as mean (95% CI) (*p < 0.05, **p < 0.01, and ***p < 0.001.).

b p-value by paired *t*-test.

#### 3.3.5 Pattern Identification for Chronic Cough Questionnaire

The pattern identification for chronic cough in our study participants was found to be widely distributed in each group of wind-cold, phlegm turbidity, liver fire, lung deficiency, and kidney yang deficiency. There were no significant differences in the pattern type distribution between the intervention and control groups. Among these five types of patterns, the wind cold type ranked first, accounting for 11 participants, followed by liver fire in nine participants (Table 2).

#### 3.3.6 Pattern Identification for GERD

Among the pattern identification of GERD, it was found that most of the participants were included in the pattern of stagnation of Liver Qi. A total of 19 participants (10 in the OJS plus SMS group and nine in the placebo group) from 30 participants were included in the category of stagnation of Liver Qi, accounting for as much as 63% of the total study participants. At baseline, the distribution of pattern identification was similar between the intervention and placebo groups, with no significant differences (Table 2).

### 3.4 Safety Assessment

Only one AE was observed during the entire trial period, which was reported to be an ankle sprain. It was determined to be mild and irrelevant to the intervention by the investigators. No significant differences were found in vital signs throughout the trial. In laboratory examinations, significant differences were found in some test results, including AST, γ-GTP, and BUN. However, all were within the normal range and were determined to have no clinical significance.

## 4 Discussion

GERD has been identified as one of the most common etiologies of chronic cough (Chung and Pavord, 2008). Multiple previous studies have focused on relieving acid reflux for the treatment of chronic cough caused by GERD. However, studies on anti-reflux therapies have failed to establish their effectiveness, and the standard treatment for cough induced by GERD remains an unresolved challenge (Kahrilas et al., 2016). This may be owing to the complex mechanisms of gastroesophageal reflux and chronic cough, which are often influenced by airway hypersensitivity and other disease processes. Thus, we believe that multi-target drugs, which affect both GI reflux and cough, will be needed as an effective therapeutic remedy for comorbidities such as GERC. In this study, we used a combination of two herbal medicines to make a multi-target medicine, which is frequently used for GERD and cough, for the treatment of GERC. To the best of our knowledge, this is the first clinical trial to explore the efficacy and safety of herbal medicines in reflux-related chronic cough. Therefore, we conducted a pilot, randomized, placebo-controlled, parallel-arm, single-center clinical trial to assess the feasibility of our study protocol. This study provides preliminary data to comprehend the mechanisms and clinical features of patients with GERC, and we anticipate that our results will be used as a scientific basis for further herbal medicine studies in GERC, as well as for the use of a combination of herbal medicines.

A total of 30 participants were enrolled in our study, 15 each in the intervention and control groups, and 25 completed the study. Various examinations including chest radiography, PFT, FeNo, and nasal endoscopy were used to exclude other potential causes of chronic cough, and those who met the diagnostic algorithm based on the inclusion and exclusion criteria were included in our study. With validated outcome measures of severity and frequency of cough, cough-specific QoL, airway reflux symptoms, and GI symptom scales, various clinical aspects of GERC were observed.

Based on the study results at baseline, the participants included in our study were revealed to have moderate to severe levels of cough, accompanied by airway reflux symptoms, and to have GI symptoms of reflux, abdominal pain, and indigestion more severe than the previously reported results from patients with GERD (Kwon et al., 2008). The total CDS was recorded to be an average of 4.05 at baseline in all of our study participants, indicating a moderate degree of cough symptoms based on the previously reported criteria of mild cough as < 3 points (Ours et al., 1999). The cough VAS score also demonstrates that our study participants had a severe degree of cough, based on the criteria of >30 points as severe cough (Smith et al., 2019). When study participants were assessed with HARQ, all except three scored higher HARQ than the cutoff value of >13 points (Morice et al., 2011). This result is consistent with other studies of chronic cough, as is well known that airway hypersensitivity is highly related to chronic cough (Morice, 2013). Additionally, unlike previous studies reporting that up to 75% of patients with GREC have no GI symptoms, we found that most of the enrolled participants in our study were suffering from reflux symptoms, even more, severe than the patients with GERD. Another interesting finding in our study was that 63% of the study participants were included in the same category of the pattern identification specified for GERD (stagnation of the Liver Qi). By comparing the results of pattern identification for chronic cough, which had found no such distribution results, we believe that this specific pattern of stagnation of Liver Qi may be an important characteristic inducing GERC in resolving the cough symptoms.

After treatment with OJS plus SMS for 6 weeks, we found significant efficacy of OJS plus SMS in the total CDS after 4 weeks and in the daytime CDS after 4 and 6 weeks compared to the placebo. As cough is generally the sole and major symptom of patients with GERC, we evaluated the CDS as the primary outcome measure in our study. We collected the CDS two times per day in daytime and nighttime, as the occurrence time of cough differs according to the causative diseases. In our study, we found better efficacy of OJS plus SMS in the daytime than in nighttime, consistent with the physiology of GREC, which has been known to induce cough during the daytime (Smith et al., 2019). Previous studies reported that this daytime cough in GERC is related to transient lower esophageal sphincter relaxation, which generally occurs during the day more than nighttime and triggers the occurrence of cough (Schoeman et al., 1995). Our findings of the superior efficacy of OJS plus SMS compared with placebo are worth considering, as there was only one study found to have benefits compared to the placebo in GERC.

Moreover, we additionally analyzed the CDS only for the participants belonging to the pattern of stagnation of Liver Qi, as a subgroup analysis. We found that OJS plus SMS had significant efficacy in all daytime, nighttime, and total CDSs at weeks four and 6 compared to the placebo. This result demonstrates that OJS plus SMS has a better therapeutic effect in the subgroup of stagnation of the Liver Qi than the other pattern types.

OJS plus SMS has also been shown to be effective in the cough VAS, which is an additional evaluation tool for the severity of cough in a wide range, from 0 to 100. We found a significant efficacy of OJS plus SMS in cough VAS after 2, 4, 6, and 8 weeks compared to the baseline. We also found that the changes in cough VAS score after treatment with OJS plus SMS for 4, 6, and 8 weeks were higher than the minimal important difference (MID) of acute cough, which was reported to be 17 mm (Lee et al., 2013). As there are no validated MIDs for chronic cough (Spinou and Birring, 2014), the MID for acute cough was used compared with our results, which were found to be decreased by 24.89, 23.67, and 21.76 points at weeks 4, 6, and 8, respectively. Although differences in cough VAS between the intervention and control groups were not found in our study, our results support the use of OJS plus SMS in clinics for patients with GERC as it had proven its clinical improvement after the treatment.

In the cough-related QoL, assessed by LCQ-K, OJS plus SMS had been revealed to have significant improvements in overall QoL scales, and each subscale of physical, psychological, and social QoL between pre-and post-treatment. We also had found clinically significant effects of OJS plus SMS after 6 weeks by exceeding the minimal clinically important differences of LCQ, previously reported as 1.7 points for LCQ total and 0.8, 0.9, and 0.8 points for physical, psychological, and social domain scores, respectively (Birring et al., 2019). In the LCQ total score of the OJS plus SMS group, it was found that only 2 weeks of treatment has clinical significance in chronic cough patients. In each subscale, we found that 2 weeks of OJS plus SMS treatment has clinical efficacy in social QoL, 4 weeks of treatment in physical QoL, and 6 weeks in psychological QoL.

When assessed for airway hypersensitivity, one of the important characteristics of GERC, we found that the majority of our study participants had symptoms of airway reflux by exceeding the cutoff value of 13, scored 24.27 in the OJS plus SMS group, and 31.33 in the placebo group at baseline. Based on our results, we also found that airway hypersensitivity is frequently observed in patients with GERC. After treatment with OJS and SMS, HARQ showed significant improvements at weeks 2, 4, and 8 compared to the baseline. However, the HARQ score was found to be over the cutoff value of 13 even at week 8, indicating that airway hypersensitivity in patients with chronic cough is not easily resolved. Although the minimal important difference (MID) of HARQ has not been established, the HARQ score after treatment was found to be lower than that previously reported in patient with GERC (Huang et al., 2016).

Lastly, as an evaluation tool for GI symptoms in patients with GERC, study participants were assessed using GSRS. Among the five symptom scales, the subscales of reflux, abdominal pain, indigestion, and constipation in our patients were revealed to be more severe than previously reported scores in patients with GERD at baseline (Kwon et al., 2008). Particularly, we had found the symptoms of reflux, abdominal pain, and indigestion were much more severe than those in patients with GERD, by which we can conclude that these are the major discomforts faced by such patients. After treatment with OJS plus SMS for 6 weeks, these three symptom scores were significantly improved compared to the baseline. The difference between the OJS plus SMS group and the placebo group after 6 weeks did not show significant results; however, OJS plus SMS improved the reflux and indigestion symptom scores more than the placebo.

Overall, OJS plus SMS improved the symptoms of cough, airway reflux, GI discomfort, and QoL in patients with GERC. Significant differences in the OJS plus SMS compared with the placebo were found in the daytime and total CDS after the treatment, and we found clinically meaningful improvements in cough score and QoL questionnaire. Our pilot study results showed the potential of OJS plus SMS in relieving the severity of cough and GI symptoms as a safe remedy.

Our study also demonstrated the feasibility of a placebo-controlled, randomized clinical trial in a clinical setting using a combination of two insurance KM granules, OJS plus SMS, in patients with GERC. We achieved successful feasibility outcomes of recruitment, completion, and adherence rates by exceeding the ratio of 80% in our pilot study. All of the planned sample sizes of 30 participants were successfully enrolled, recording a recruitment rate of 100%, and 25 out of 30 participants completed the trial, resulting in a completion rate of 83%. The adherence rate of the trial visit was 93%, as one participant dropped out at visit 3, three at visit 4, and one at visit 5. Based on these results, it is assumed that the overall study design of inclusion/exclusion criteria, number of trial visits, duration of trial drug administration, and follow-up period were appropriate for our study. Moreover, it was found that the primary outcome of the CDS was appropriate for detecting the efficacy of OJS plus SMS in patients with GERC. We found significant differences in CDS between the OJS plus SMS group and the placebo group, and also revealed that the difference was mostly due to the improvement of the daytime cough. As CDS was assessed twice daily and at night, it was useful to discriminate the causative diseases of chronic cough and to determine the relationship with GERD. Other outcome measures of cough, HRQL, and GI symptoms also showed a positive trend and clinical importance of OJS plus SMS, allowing it to be used in further studies as well.

However, we need to consider some of the limitations of our study when developing our next trial protocol. First, despite its successful recruitment rate, we need to consider the recruitment duration for our next trial, as it took almost 2.5 years to recruit 30 participants. It will be necessary to plan a sufficient period for participant recruitment, and the trial should be conducted as a multicenter trial in our next large-scale trial. Second, outcome measures should be supplemented to detect the efficacy of OJS plus SMS for GERC. Although CDS had successfully detected the effect size of OJS plus SMS in our study, we can consider using it as a daytime CDS and nighttime CDS separately, and not as a total CDS for the primary outcome in our next trial. As the occurrence time of cough provides important information for understanding the physiology of cough, each of the daytime and nighttime cough symptom scores will be more sensitive to GERC than the total score. Additionally, the objective outcomes of reflux syndrome can be considered to investigate the mechanisms and subtypes of GERC. Recently, multichannel intraluminal impedance combined with pH monitoring has been used to discriminate between acid and non-acid reflux. Although it is not the standard instrument for GERC, it can provide more detailed information to understand the pathology of cough reflux syndrome. Third, we did not consider some factors that were reported to affect cough outcomes, including lifestyle modifications or weight. In previous studies of GERC, studies including these factors showed better cough outcomes (Kastelik et al., 2005; Ojoo et al., 2013). These lifestyle modifications will need to be controlled for participants in our next study design. Fourth, the typical features of reflux symptoms, such as heartburn and regurgitation, were not used as inclusion/exclusion criteria or as stratification criteria. These features are important, as the effects of medicines respond differently between patients with and without reflux symptoms. This can be seen in the use of PPIs, which are only recommended for patients with reflux symptoms. Thus, we will need to stratify study participants according to reflux symptoms to evaluate the differences in respondence between the two groups. Fifth, there was a strong placebo effect on the outcomes of cough and GI symptoms. This phenomenon has been frequently observed in previous studies with GERC (Kahrilas et al., 2016) as well as in studies of cough. There was only one study that reported the benefits of cough compared to the placebo, which was even the study conducted on patients with laryngopharyngeal reflux patients (Pawar et al., 2007). Additionally, the placebo effects in cough medicines have been reported to be powerful because several effects are related to the efficacy of the medicine, such as a physiological effect (taste of the medicine), a nonspecific effect (natural recovery), and a true place effect (psychological effect) (Eccles, 2020). Although we found that OJS plus SMS showed better efficacy compared to the placebo in the CDS, the placebo also showed considerable improvements in the severity of cough and GI symptoms. We can consider using tablets or capsules to avoid placebo effects, as is recommended for minimizing the placebo effects in cough medicine. Lastly, the sample size was not sufficient to confirm the efficacy of our intervention. As it was a pilot study, the major goal of this study was to assess the feasibility of the study protocol, and we have shown preliminary data of OJS plus SMS in the treatment of GERC. Our pilot study results will be used to calculate the effect size and sample size for the next trial to confirm the safety and efficacy of OJS plus SMS in GERC.

In conclusion, we confirmed the feasibility of our trial design and found that OJS plus SMS was safe and effective for the treatment of GERC. We have presented the possibility of herbal medicines in GERC for the first time, particularly by using a combination of two herbal medicines being used for different indications. Additionally, the role of the pattern type of stagnation of the Liver Qi in the GERC is worth considering, as we found that OJS plus SMS responded best in that pattern type. Along with our study results and some limitations, a protocol for a well-designed, large-scale confirmatory trial is needed to confirm the safety and efficacy of OJS plus SMS in GERC patients.

## Data Availability

The original contributions presented in the study are included in the article/Supplementary Material, further inquiries can be directed to the corresponding authors.

## Supplementary Material

Supplementary Table S1 Cough diary score in OJS plus SMS group and placebo group.
