# Supplementary material for: Efficacy and Safety of Ojeok-San Plus Saengmaek-San for Gastroesophageal Reflux-Induced Chronic Cough: A Pilot, Randomized, Double-Blind, Placebo-Controlled Trial
Source: Front Pharmacol. 2022 Mar 1;13:787860. doi: 10.3389/fphar.2022.787860 (PMC8923584; doi:10.3389/fphar.2022.787860)
Supplement: Supplementary file 1 [file Table1.docx]

| ***Supplementary data*** | | | | | | |
| --- | --- | --- | --- | --- | --- | --- |
| **Table S1. Cough Diary Score in OJS plus SMS group and Placebo group** | | | | | | |
| **Cough Diary**  **Score** | **OJS plus SMS (n=15)** | |  | **Placebo (n=15)** | | *P* value^b^  (OJS + SMS *versus* placebo) |
|  | Mean (95% CI) | *P* value^a^  (within group) |  | Mean (95% CI) | *P* value^a^  (within group) |  |
| **Daytime** | | | | | | |
| Baseline | 3.93 (3.44, 4.42) |  |  | 4.13 (3.40, 4.86) |  |  |
| Week 2 | 2.75 (2.24, 3.27) | <.0001*** |  | 3.20 (2.40, 4.00) | 0.0082** | 0.3807 |
| Week 4 | 1.70 (1.05, 2.35) | <.0001*** |  | 2.85 (2.04, 3.65) | 0.0017** | 0.0191* |
| Week 6 | 1.49 (0.87, 2.10) | <.0001*** |  | 2.48 (1.79, 3.17) | <.0001*** | 0.0267* |
| Week 8 | 1.90 (1.24, 2.57) | <.0001*** |  | 2.06 (1.34, 2.77) | <.0001*** | 0.9076 |
| **Nighttime** | | | | | | |
| Baseline | 3.89 (3.36, 4.42) |  |  | 4.22 (3.50, 4.94) |  |  |
| Week 2 | 2.75 (2.17, 3.33) | 0.0005*** |  | 3.25 (2.44, 4.05) | 0.0080** | 0.4773 |
| Week 4 | 1.86 (1.23, 2.48) | <.0001*** |  | 2.83 (2.01, 3.66) | 0.0009*** | 0.0766 |
| Week 6 | 1.64 (1.02, 2.25) | <.0001*** |  | 2.50 (1.79, 3.20) | <.0001*** | 0.1063 |
| Week 8 | 1.92 (1.26, 2.57) | <.0001*** |  | 2.06 (1.37, 2.76) | <.0001*** | 0.9851 |
| **Total** | | | | | | |
| Baseline | 3.91 (3.44, 4.37) |  |  | 4.18 (3.46, 4.90) |  |  |
| Week 2 | 2.75 (2.20, 3.29) | <.0001*** |  | 3.22 (2.43, 4.01) | 0.0072** | 0.4396 |
| Week 4 | 1.80 (1.18, 2.42) | <.0001*** |  | 2.83 (2.02, 3.65) | 0.0010** | 0.0427* |
| Week 6 | 1.57 (0.94, 2.19) | <.0001*** |  | 2.51 (1.82, 3.20) | <.0001*** | 0.0555 |
| Week 8 | 1.90 (1.20, 2.60) | <.0001*** |  | 2.07 (1.36, 2.77) | <.0001*** | 0.931 |
| ^a^ p-value by paired t-test ^b^ p-value by Analysis of covariance (ANCOVA); OJS, Ojeok-san; SMS, Saengmaek-san; Data are presented as mean (95% CI) (*p < 0.05, **p < 0.01, and ***p < 0.001.) | | | | | | |
